# A software package for immunologists to learn simulation modeling

**DOI:** 10.1186/s12865-019-0321-0

**Published:** 2020-01-02

**Authors:** Andreas Handel

**Affiliations:** Department of Epidemiology and Biostatistics and Health Informatics Institute and Center for the Ecology of Infectious Diseases, The University of Georgia, Athens, GA USA

**Keywords:** Mechanistic simulation models, Teaching software, R package

## Abstract

**Background:**

As immunology continues to become more quantitative, increasingly sophisticated computational tools are commonly used. One useful toolset are simulation models. Becoming familiar with such models and their uses generally requires writing computer code early in the learning process. This poses a barrier for individuals who do not have prior coding experience.

**Results:**

To help reduce this barrier, I wrote software that teaches the use of mechanistic simulation models to study infection and immune response dynamics, without the need to read or write computer code. The software, called *Dynamical Systems Approach to Immune Response Modeling (DSAIRM)*, is implemented as a freely available package for the R programming language. The target audience are immunologists and other scientists with no or little coding experience. DSAIRM provides a hands-on introduction to simulation models, teaches the basics of those models and what they can be used for. Here, I describe the DSAIRM R package, explain the different ways the package can be used, and provide a few introductory examples.

**Conclusions:**

Working through DSAIRM will equip individuals with the knowledge needed to critically assess studies using simulation models in the published literature and will help them understand when such a modeling approach might be suitable for their own research. DSAIRM also provides users a potential starting point towards development and use of simulation models in their own research.

## Background

Immunological data continues to increase rapidly in quantity, quality and complexity. Concomitant with this increase in data acquisition is an increased sophistication in the way data are analyzed. Modern approaches include many often complex analytical and computational tools. One useful set of such tools are mechanistic simulation models. Several books and review articles describe applications of such computational models to study infection and immune response (see e.g. [1–7]). While these are good resources, reading alone is often not sufficient for thorough learning. *Active learning* often leads to better outcomes [8–10]. To learn about computational simulation models, directly engaging with them is an obvious method that can facility such active learning. However, this generally requires writing computer code.

The need to write code can pose a significant barrier for individuals who do not have prior coding experience. To reduce this barrier, I wrote software that allows individuals to obtain an introduction to simulation modeling of within-host infection and immune dynamics, without the need to read or write computer code. The software, called *Dynamical Systems Approach to Immune Response Modeling (DSAIRM)*, is implemented as a freely available package for the widely used R programming language. The DSAIRM package is meant for immunologists and other bench scientists who have little or no coding and modeling experience and who are interested in learning how to use systems simulation models to study within-host infection and immune response dynamics.

By engaging with DSAIRM, users will be provided with a hands-on introduction to simulation models and will learn the basics of how those models work and what they can be used for. This will allow users to understand modeling results reported in the literature. It will also allow users to determine if modeling is a useful tool for them and if so, DSAIRM provides a starting point to using such models for their own research.

This paper describes the DSAIRM package and provides a quick start guide and some illustrating examples.

## Implementation

The package consists of simulations (in the following referred to as apps) that allow the exploration and learning of different infection and immune response modeling topics. The underlying models are implemented as either deterministic or stochastic compartmental dynamical models (ordinary differential equations, discrete-time models, or their stochastic counterparts). While some mathematical details are provided for the models, all are described in plain language such that users do not need to be familiar with differential equations or other advanced mathematics to use DSAIRM and learn the material. A graphical user interface is wrapped around each simulation. This allows exploration of models and topics without the need to read or write any computer code. Each app includes a detailed description of the model and topic being covered, and a list of tasks a user should try. The documentation included in DSAIRM strives to be detailed enough to serve as a stand-alone learning environment. References are provided with each app for further reading and learning. The package is structured in a modular way to allow users a fairly seamless transition toward more flexibility and power by directly interacting with and modifying the underlying simulations. This comes with a gradual increase in the required amount of coding. The different ways of interacting with and progressing through the package are described below.

## Results

### Installing and running the package

**Fig. 1:**
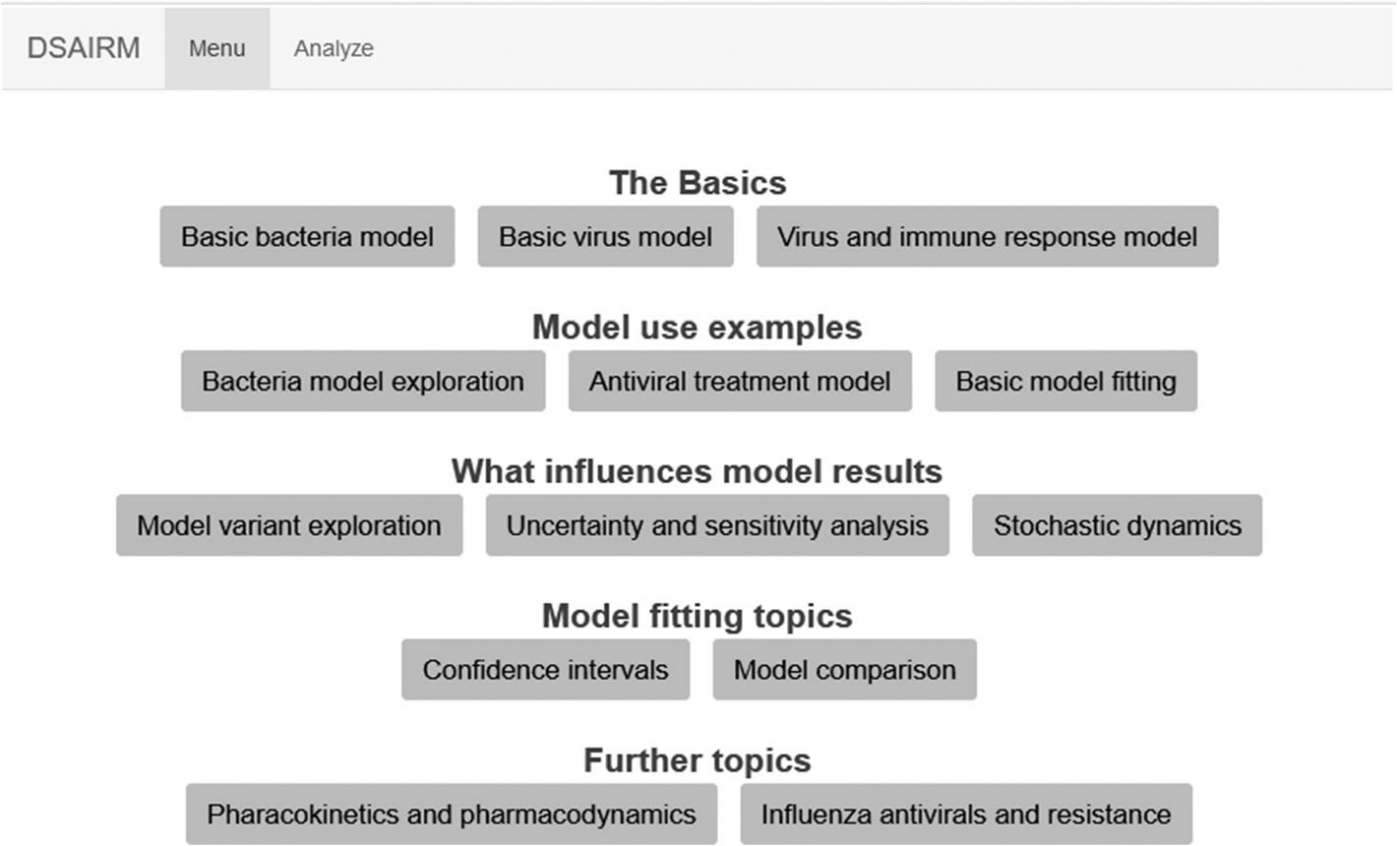
Main menu of the DSAIRM package. From this menu, the user can access and interact with each simulation app. Once finished, the user can exit the menu and shut down R. No reading or writing of code beyond the single command starting the graphical menu is required

Package installation is a one-time process, unless R itself is being reinstalled. The package depends on other packages, which will be automatically installed as needed. At every new start of R, the package needs to be loaded before it is ready for use. The following are quick-start instructions:

- Install R from https://cran.r-project.org/
- Optional, recommended: Install RStudio from https://www.rstudio.com/
- Open R/Rstudio, install the package by typing install.packages(‘DSAIRM’) into the R console. (This will also install packages required by DSAIRM).
- Load the package with library(‘DSAIRM’).
- Call the main menu by typing dsairmmenu() into the R console. A graphical interface showing the main menu (Fig. 1) should open in the browser.
- You are ready to explore!

### Using the package

The different ways DSAIRM can be used are described in the following sections. All users are expected to start with *Level 1*. Further progression to *Levels 2 and 3* is optional and based on needs and interests.

### Level 1: interactive use through the graphical user interface

Using the graphical interface to interact with and explore the models and topics is the main intended use of DSAIRM. To get to the graphical interface, load the package and call the main menu as described above. This will bring up a menu (Fig. 1) from which one can select each simulation app.

**Fig. 2:**
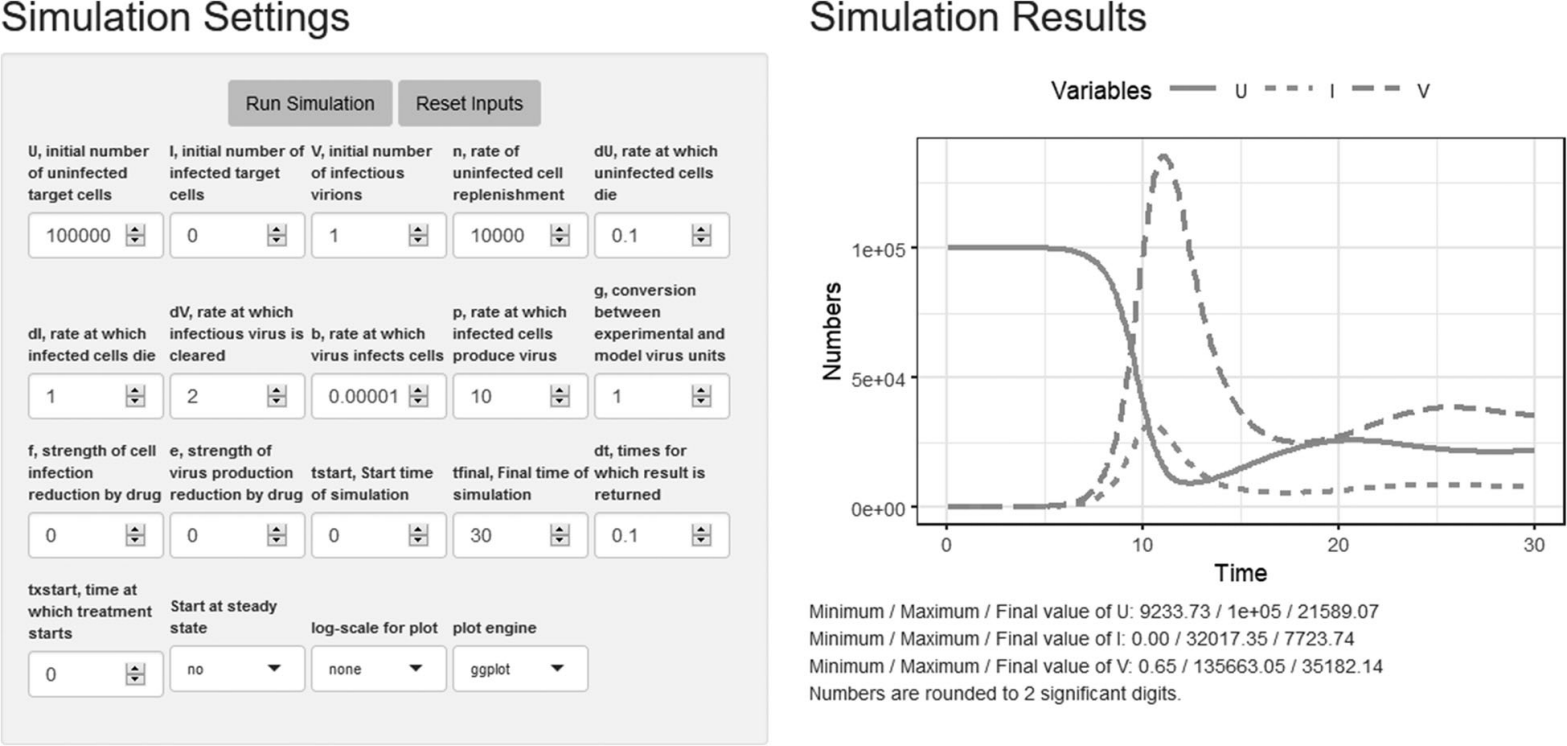
Graphical interface for the Antiviral treatment model app. Inputs are on the left, outputs in the form of graphs and useful numbers (e.g. maximum of each variable during the simulation) are provided on the right

Each app has input boxes on the left which allow one to specify model parameters and other settings. To the right, results are displayed as text and graphs. See Fig. 2 for an example.

**Fig. 3:**
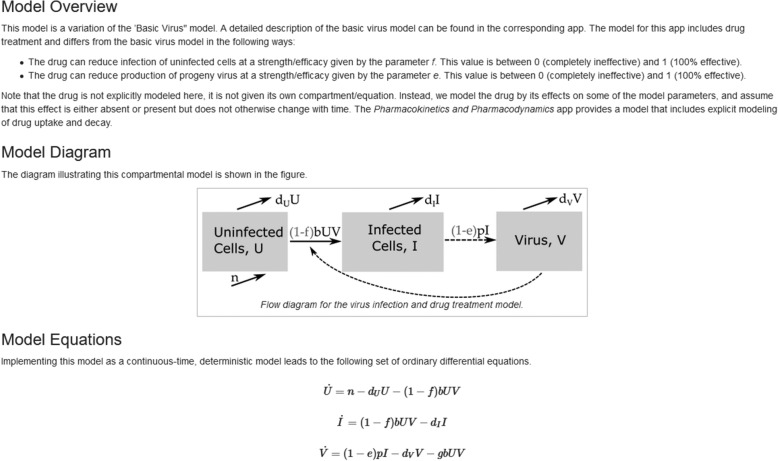
Part of the Model documentation tab for the Antiviral treatment model app. The app in which a model is first used includes a detailed verbal explanation of all variables and all processes that are part of the model. This is followed by a model diagram and model equations. If a model is re-used in subsequent apps, as is the case here, the previous app is referenced and the model description is shortened

Below the input and output areas are several tabs which contain detailed information for each app. The *Overview* section briefly states the topic covered by the app. The *Model* section describes the model in detail and provides additional background information on specific modeling topics. Where applicable, the model diagram and equations are shown. Figure 3 shows a screenshot of part of the *Model* section for one of the apps. The *What to do* section contains a list of suggested tasks. Together, the *Model* and *What to do* sections are the main teaching components of each app. By working through those sections, the user will be able to get a good understanding of what the model is and what it does and will learn about important modeling concepts and topics. The *Further Information* section lists the underlying simulation functions used in the app, as well as provides pointers to the literature for additional reading on the covered topic.

After exploring an app, return to the main menu to choose another app to explore. Once done, exit the main menu and close the R session. At this level of interaction with the models, no code needs to be read or written. One can fully focus on exploring and learning about several infection and immune response models and gain an understanding of the strengths, limitations and main use cases for these kinds of models. This should provide a good understanding of results from such models reported in the research literature. It will also allow teach the user if this type of modeling approach might be suitable for their specific research systems and questions.

This stage of DSAIRM use might provide enough insight into those types of models, and a user might want to leave it at that. Alternatively, if the user wants to implement simulation models for their own research, they could proceed to the next levels of engagement with DSAIRM. Of course, at this stage it is also always possible to find a modeling expert and start a collaboration, which is the approach we suggest for most individuals.

### Level 2: directly interacting with the simulation functions

To continue the modeling journey, it is possible to use the simulations provided in DSAIRM in a more direct manner, without the graphical user interface. This provides more flexibility at the cost of having to write a limited amount of code. The *Further Information* section of each app provides the name of the underlying simulation function that one can interact with directly.

Consider as example the first app, called “Basic Bacteria Model”. This model has 2 underlying simulator functions, one that runs a discrete-time model called simulate_basicbacteria_discrete and one that runs a continuous, differential equation model called simulate_basicbacteria_ode. Assume we are interested in the latter. The documentation for this function provides details regarding model inputs and outputs. This documentation can be accessed by typing the following into the R console (the DSAIRM package needs to be loaded for this to work):

**help**('simulate_basicbacteria_ode')

The help file explains that one can run the simulation by specifying initial number of bacteria and immune response strength, the different model parameters, as well as some time values. For most apps, time units for the model are determined by the time unit chosen to express the parameters in. Each model input has a default value, which is used if the model is called without providing specified inputs. One can overwrite those default settings. For instance, the following line of code calls the simulator and overwrites the default values for the rate at which bacteria grow, *g*, and the rate at which the immune response is induced and grows, *r*, while using the default values for the remainder (this is equivalent to setting different inputs through the graphical interface in level 1):

result <-**simulate_basicbacteria_ode**(g = 0.5, r = 0.002)

Calling the simulation function executes the underlying model. For this simulation, the function returns time-series for each of the variables that are tracked, namely bacteria and immune response. Not all simulation functions return time series. For every simulation function, the help file explains what is returned. One can further process those returned results. A basic plot of bacterial load as function of time can be produced with this line of code (resulting plot not shown):

**plot**(result**$**ts[,"time"],result**$**ts[,"B"],xlab='Time',ylab='Bacterial Load',type='l')

**Fig. 4:**
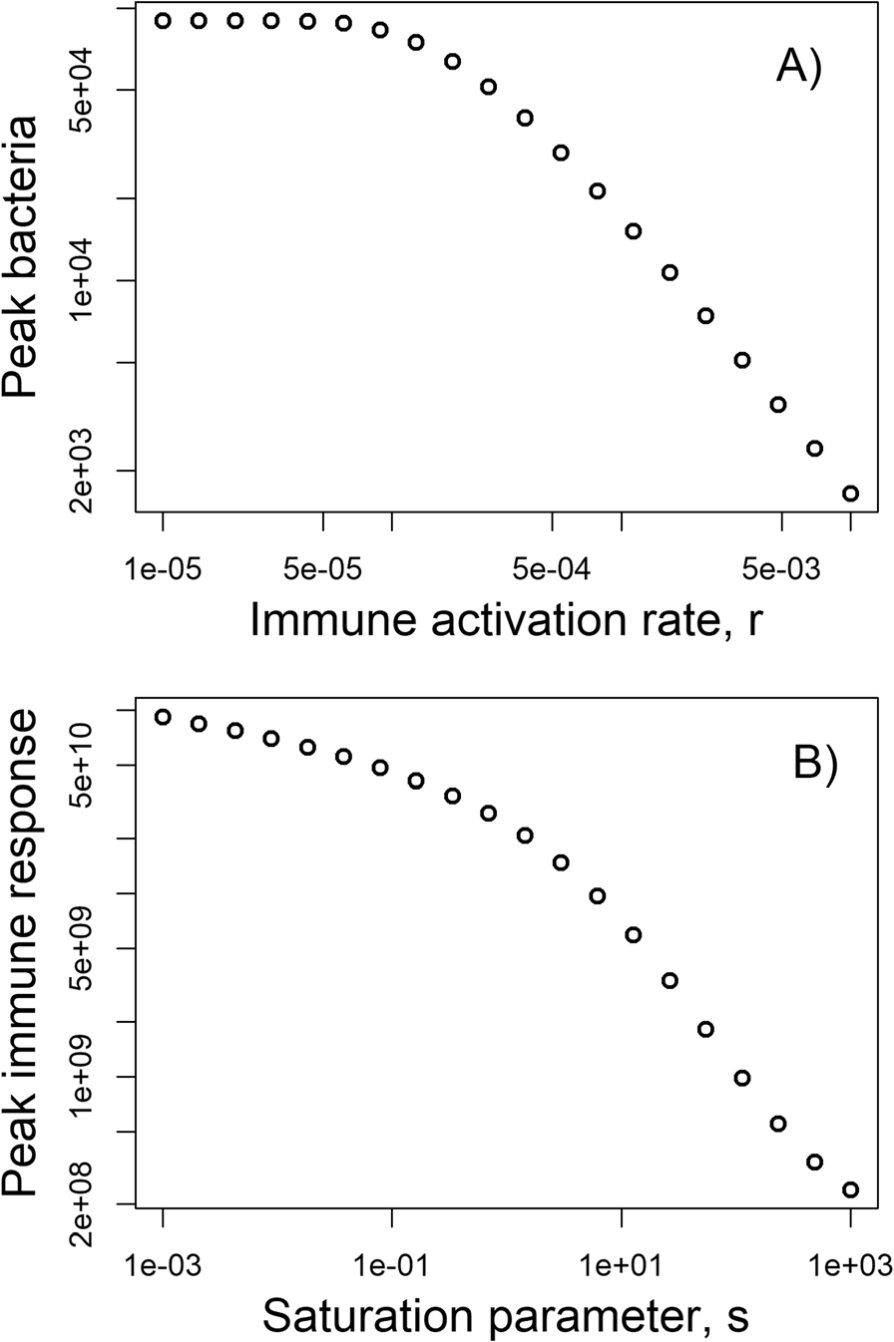
Examples for level 2 and level 3 use of the package. **a** Peak bacterial load as function of immune response activation rate for the model described in the level 2 section. **b** Maximum of the immune response as a function of the saturation parameter for the model described in the level 3 section

Calling the simulation functions without using the graphical interface makes model exploration more efficient and flexible. Assume we wanted to determine how some parameter influences the outcome of the model, for instance how the peak bacterial load changes with the immune response activation rate, *r*, (while keeping all other parameters fixed). With the graphical interface, one needs to manually set different parameter values for *r*, run the model for each parameter value and write down the peak bacterial load. This can be automated by calling the simulation function directly. The following lines of code show how this can be achieved. A loop is run over different *r* values, for each *r* value the simulation is run, and the peak bacterial load is recorded. At the end, this quantity as a function of the immune activation rate is plotted. This plot is shown in Fig. 4a.

*#values for immune activation rate, r, for which to run the simulation*

rvec = 10**^seq**(**-**5,**-**2,length=20)

*#this variable will store the peak values for each r*

Bpeak =**rep**(0,**length**(rvec))

**for**(n**in**1**:length**(rvec))

{

*#run the simulation model for different values of r*

*#for simplicity, all other inputs are kept at their defaults*

result <-**simulate_basicbacteria_ode**(r = rvec[n])

*#peak bacterial load for each value of r*

Bpeak[n] <-**max**(result**$**ts[,"B"])

}

*#plot final result*

**plot**(rvec,Bpeak,type='p',xlab='Immune activation rate, r',ylab='Peak bacterial load',log='xy')

This example illustrates how, with a few lines of extra R code, one can explore the models much more efficiently and flexibly than would be possible through the graphical interface. The trade-off is that one needs to write some code and thus acquire some familiarity with the basics of R.

### Level 3: modifying the simulation functions

While the *Level 2* approach allows one to use the models in a way that would be hard or impossible using the graphical interface, there is still one major constraint. Namely the *Level 2* approach only allows analysis of existing DSAIRM simulation models. While it might be possible that one of these models is applicable to a specific research question, it is much more likely that a model that is somewhat different from those that come with DSAIRM is needed. In that case, it is possible to use the code for one of the DSAIRM models as starting point and modify it as needed.

Copies of all simulator functions can be downloaded directly from the main menu. The code for each simulator function is (hopefully) well documented. However, the level of coding and R knowledge required to modify the functions is higher than that required to use the functions as described in *Level 2*. To provide an example, assume a system for which the basic bacteria ODE model in DSAIRM is almost, but not quite applicable. Instead of the model provided by DSAIRM, a model is needed in which the immune system growth term includes saturation at some maximum rate when bacterial load is high. This can be accomplished by a change of the term *rBI* to *rBI/(B + s)*. (See the documentation for this app for an explanation of each model term). This change leads to a growth at rate *rB* proportional to the number of bacteria if *B* is smaller than some threshold parameter *s*, and turns into a growth at fixed maximum rate *r*, independent of bacterial load, if *B* is larger than *s*.

To implement this, one can modify the code for this model, which is in the file simulate_basicbacteria_ode.R. (To make things easy, the name of a. R file containing the code and the name of the function itself are the same.) After finding the file, making a copy and renaming it (here called mysimulator.R), one can edit the model from the one provided to the one needed by making the following modifications to the code:

old:

simulate_basicbacteria_ode <-**function**(B = 10, I = 1, g = 1, Bmax = 1e+05, dB = 0.1, k = 1e-06, r = 0.001, dI = 1, tstart = 0, tfinal = 30, dt = 0.05)

new:

mysimulator <-**function**(B = 10, I = 1, g = 1, Bmax = 1e+05, dB = 0.1, k = 1e-06, r=1e3, dI=1, tstart = 0, tfinal = 30, dt = 0.05, s=1E3)

Note the changed default value for *r* to ensure the immune response is sufficiently triggered.

old:

pars =**c**(g=g,Bmax=Bmax,dB=dB,k=k,r=r,dI=dI)

new:

pars =**c**(g=g,Bmax=Bmax,dB=dB,k=k,r=r,dI=dI,s=s)

old:

dBdt = g*B*(1**-**B**/**Bmax)**-**dB*B**-**k*B*I

dIdt = r*B*I**-**dI*I

new:

dBdt = g*B*(1**-**B**/**Bmax)**-**dB*B**-**k*B*I

dIdt = r*B*I**/**(s**+**B)**-**dI*I

With these changes made, one can investigate the behavior of this new model. For instance, one can explore how different values of the saturation parameter, *s*, impact the maximum level of the immune response. This requires a slight modification of the code shown above in *Level 2* as follows, the resulting plot is shown in Fig. 4b.

*#initialize the new function*

*#it needs to be in the same directory as this code*

**source**('mysimulator.R')

*#values of saturation parameter to explore*

svec = 10**^seq**(**-**3,3,length=20)

*#this will record the maximum immune response level*

Ipeak =**rep**(0,**length**(svec))

**for**(n**in**1**:length**(svec))

{

*#run the simulation model for different values of s*

*#for simplicity, all other inputs are kept at their defaults*

result <-**mysimulator**(s = svec[n])

*#record max immune response for each value of s*

Ipeak[n] <-**max**(result**$**ts[,"I"])

}

**plot**(svec,Ipeak,type='p',xlab='Saturation parameter, s',ylab='Peak immune response',log='xy')

Using one of the provided simulator functions as starting point and modifying it is likely easier than having to write a new model completely from scratch. Eventually, with more coding experience, the user gains (almost) unlimited flexibility regarding the models they can create, of course at the cost of having to write increasingly more R code. The limit is only what can be accomplished using the R programming language and one’s ability and interest in writing customized code.

### Beyond level 3

The source code for DSAIRM is public and available on GitHub [11]. It is quite likely that there are still bugs and typos in the package and its documentation. Submission of bug reports, feature requests, or any other feedback is very welcome. The preferred mode of such input is through the package’s GitHub site. Contributions of new apps or other enhancements are also very welcome. More information is provided on the DSAIRM website [11].

Depending on the modeling task, it might be suitable to go beyond what DSAIRM provides. Specialized software suitable for implementing more complex models exists. SIMMUNE allows the graphical building and analysis of rather detailed spatial models [12]. IMMSIM is another software that allows implementation and simulation of detailed immune response models [13]. ENISI focuses on modeling immunology for enteric pathogens [14]. More generalist software packages such as COPASI [15], BioNetGen [16] or Berkeley Madonna [17] also allow implementation and analysis of within-host and immune system models. Monolix [18] allows analysis and fitting of similar models with a focus on drug development. These are some examples of software suitable for immunology, others exist [19]. Some of these software packages require coding, others allow a graphical approach to model building and analysis.

The main difference between those software packages and DSAIRM is that DSAIRM’s focus is on teaching and learning and providing a gentle introduction to simulation models. As such, models are kept simple and presented in a user-friendly, teaching-focused manner. While the option to access the underlying code and modify it exists, this will require coding in R and thus has all the general advantages and disadvantages of the R language. While R is flexible and powerful, for certain tasks other software like the ones just mentioned might be more suitable.

## Conclusions

I described DSAIRM, an R software package which allows individuals to learn the basics of mechanistic simulation modeling applied to infection and immune response dynamics. The primary goal for this software is to provide immunologists and other bench scientists with a hands-on, interactive introduction to the basics and uses of simulation modeling, without having to read or write code, or knowing any advanced mathematics. At the same time, the package is designed to allow easy advancement toward increased flexibility in addressing questions of interest, with a concomitant (gentle) increase of required coding. Users have the option of customizing the provided models to their specific needs and are eventually able to tap into all functionality available within the powerful R language eco-system. My hope is that this package will continue to grow and become a widely used and useful resource for individuals interested in learning about and potentially using such modeling approaches as part of their research.

## Data Availability

All materials described in this article are freely available on the package’s GitHub site.

## Abbreviation

DSAIRM: Dynamical Systems Approach to Immune Response Modeling
